# CRP1 Protein: (dis)similarities between *Arabidopsis thaliana* and *Zea mays*

**DOI:** 10.3389/fpls.2017.00163

**Published:** 2017-02-15

**Authors:** Roberto Ferrari, Luca Tadini, Fabio Moratti, Marie-Kristin Lehniger, Alex Costa, Fabio Rossi, Monica Colombo, Simona Masiero, Christian Schmitz-Linneweber, Paolo Pesaresi

**Affiliations:** ^1^Dipartimento di Bioscienze, Università degli studi di MilanoMilano, Italy; ^2^Max-Planck-Institut für Molekulare PflanzenphysiologiePotsdam-Golm, Germany; ^3^Molecular Genetics, Institute of Biology, Humboldt University of BerlinBerlin, Germany; ^4^Dipartimento di Biotecnologie Mediche e Medicina Traslazionale, Università degli studi di MilanoMilano, Italy; ^5^Centro Ricerca e Innovazione, Fondazione Edmund MachSan Michele all’Adige, Italy; ^6^Dipartimento di Scienze Agrarie e Ambientali - Produzione, Territorio, Agroenergia, Università degli studi di MilanoMilano, Italy

**Keywords:** PPR, anterograde signaling, chloroplast, biogenesis, RNA metabolism

## Abstract

Biogenesis of chloroplasts in higher plants is initiated from proplastids, and involves a series of processes by which a plastid able to perform photosynthesis, to synthesize amino acids, lipids, and phytohormones is formed. All plastid protein complexes are composed of subunits encoded by the nucleus and chloroplast genomes, which require a coordinated gene expression to produce the correct concentrations of organellar proteins and to maintain organelle function. To achieve this, hundreds of nucleus-encoded factors are imported into the chloroplast to control plastid gene expression. Among these factors, members of the Pentatricopeptide Repeat (PPR) containing protein family have emerged as key regulators of the organellar post–transcriptional processing. PPR proteins represent a large family in plants, and the extent to which PPR functions are conserved between dicots and monocots deserves evaluation, in light of differences in photosynthetic metabolism (C3 vs. C4) and localization of chloroplast biogenesis (mesophyll vs. bundle sheath cells). In this work we investigated the role played in the process of chloroplast biogenesis by At5g42310, a member of the Arabidopsis PPR family which we here refer to as *At*CRP1 (Chloroplast RNA Processing 1), providing a comparison with the orthologous *Zm*CRP1 protein from *Zea mays*. Loss-of-function *atcrp1* mutants are characterized by yellow-albinotic cotyledons and leaves owing to defects in the accumulation of subunits of the thylakoid protein complexes. As in the case of *Zm*CRP1, *At*CRP1 associates with the 5′ UTRs of both *psaC* and, albeit very weakly, *petA* transcripts, indicating that the role of CRP1 as regulator of chloroplast protein synthesis has been conserved between maize and Arabidopsis. *At*CRP1 also interacts with the *petB-petD* intergenic region and is required for the generation of *petB* and *petD* monocistronic RNAs. A similar role has been also attributed to *Zm*CRP1, although the direct interaction of *Zm*CRP1 with the *petB-petD* intergenic region has never been reported, which could indicate that *At*CRP1 and *Zm*CRP1 differ, in part, in their plastid RNA targets.

## Introduction

In land-plants, nuclear-encoded pentatricopeptide repeat (PPR) containing proteins constitute a large family, which regulates organelle gene expression at the RNA level (Lurin et al., 2004; O’Toole et al., 2008; Barkan and Small, 2014). They are, indeed, a major constituent of the genome-coordinating anterograde signaling pathway that evolved to adapt the expression of the organellar genomes in response to endogenous and environmental stimuli that are perceived by the nucleus (Woodson and Chory, 2008).

A typical PPR motif is characterized by a degenerate 35-amino acid repeat that folds into two antiparallel alpha helices (Small and Peeters, 2000). PPR proteins contain a tandem array of 2–30 PPR motifs, which stack together to form a superhelix with a central groove that allows the protein to bind RNA (Lurin et al., 2004; Rivals et al., 2006). According to the characteristics of their repeats, PPR proteins are generally classified into P and PLS sub-families. The P-type proteins are implicated in the determination and stabilization of 5′ and/or 3′ RNA termini, RNA splicing and translation of specific RNAs in chloroplasts and mitochondria, while PLS-type proteins are generally involved in RNA editing (Barkan and Small, 2014). Higher plants harbor several hundreds of PPR proteins, which generally have distinct, non-redundant functions in organelle biogenesis, plant growth and development and adaptation to environmental cues (Barkan and Small, 2014; Manna, 2015), as revealed by the high number of *ppr* mutants with distinct phenotypes. This is due to their ability to recognize primary RNA sequences, with each protein having different target sites, thus implying that the elucidation of the primary role of each PPR protein is greatly facilitated by the identification of its RNA targets.

The detection of few native PPR-RNA interactions through RNA immunoprecipitation on microarray (RIP-Chip) analyses and *in vitro* binding assays using PPR recombinant proteins, together with PPR crystal structures indicate that PPR proteins bind their cognate RNA targets in a sequence specific manner (Meierhoff et al., 2003; Schmitz-Linneweber et al., 2005, 2006; Williams-Carrier et al., 2008; Yin et al., 2013; Okuda et al., 2014; Shen et al., 2016). The code describing how PPR proteins recognize specific nucleotides of their RNA targets relies primarily on two amino acids that are within a single PPR motif, specifically the fifth residue in the first helix and the last residue on the loop interconnecting adjacent motifs (Barkan et al., 2012; Yin et al., 2013; Cheng et al., 2016). However, the current understanding of the code does not allow accurate large-scale computational predictions of PPR targets (Takenaka et al., 2013; Kindgren et al., 2015; Hall, 2016; Harrison et al., 2016). Predictive power is constrained by the fact that the code is degenerate and by the low accuracy of current methods used for the identification of PPR domains, which in turn leads to mismatches in the amino acid/nucleotide alignments. However, a more robust annotation of PPR domains has recently been conducted and made available at the PlantPPR database^1^ (Cheng et al., 2016). Furthermore, more PPR-RNA interactions as well as crystal structures of PPR-RNA complexes need to be characterized in different species in order to improve the understanding of the code. This would also help to determine if the amino acid sequences of the PPR domains coevolved with the nucleotide sequences of their RNA targets and ultimately to determine whether there is functional conservation of PPR proteins among land plants.

The function of PPR proteins, and more generally the function of the nuclear gene complement involved in organellar RNA metabolism, have been primarily studied in maize, since the large seed reserves of maize support rapid heterotrophic growth of non-photosynthetic mutants and provide ready access to non-photosynthetic tissues for molecular biology and biochemical studies (Belcher et al., 2015). However, the degree of functional conservation of PPR proteins between maize and other species, including *Arabidopsis thaliana*, has yet to be investigated. The question is of particular interest since the elaboration of the thylakoid membrane system and the biogenesis of the multi-subunit photosynthetic complexes appear to have major differences between monocotyledonous and dicotyledonous plants (Pogson et al., 2015). Indeed in maize, and more generally in monocots, the process of chloroplast development from the proplastid to functional chloroplasts can be observed as a gradient along the leaf blade, whereas in dicots, such as *Arabidopsis thaliana*, the development of chloroplasts differs between developmental stages, plant organs – i.e., chloroplast development is different in cotyledons and leaves – and plant tissues (Pogson and Albrecht, 2011; Jarvis and Lopez-Juez, 2013).

The majority of PPR proteins are conserved at sequence level between dicots (Arabidopsis) and monocots (rice) (O’Toole et al., 2008). Orthologous pairs can readily be identified and in a number of cases, primary sequence conservation can be traced back to the roots of all embryophytes (O’Toole et al., 2008). As a matter of fact, functional differences between orthologous PPR proteins of maize and Arabidopsis have been observed. For example, the molecular phenotypes resulting from loss of the orthologous PPR proteins ATP4 (maize) and SVR7 (Arabidopsis) differ substantially (Liu et al., 2010; Zoschke et al., 2012, 2013a,b), as do the molecular defects in maize and Arabidopsis mutants lacking the PGR3 protein (Yamazaki et al., 2004; Cai et al., 2011; Belcher et al., 2015). Thus, the extent to which lessons on PPR proteins learnt from maize can be extrapolated to dicots, such as Arabidopsis, and more broadly to other organisms, needs further investigation.

In this context, we investigated here the function of and identified the RNA targets of the PPR protein At5g42310 from *Arabidopsis thaliana*, that shares high similarity with the well-characterized CRP1 (Chloroplast RNA Processing 1) protein from maize (*Zm*CRP1), and which we here refer to as *At*CRP1. Our findings indicate that *At*CRP1, like the orthologous *Zm*CRP1 (Barkan et al., 1994; Fisk et al., 1999; Schmitz-Linneweber et al., 2005), is essential for plant autotrophy since it plays a direct role in the accumulation of the cytochrome *b_6_/f* (Cyt *b_6_/f*) complex and of the PsaC subunit of photosystem I (PSI). Furthermore *At*CRP1, similarly to *Zm*CRP1, is required for the accumulation of *petB* and *petD* monocistronic RNAs, indicating that the functional roles of CRP1 proteins are highly conserved between monocots and dicots.

## Materials and Methods

### Plant Material and Growth Conditions

*Arabidopsis thaliana atcrp1-1* (SALK_035048) (Alonso et al., 2003) and *atcrp1-2* (SAIL_916A02) (Sessions et al., 2002) T-DNA insertion lines were identified by searching the T-DNA Express database^2^. For promoter analyses, the putative *AtCRP1* promoter region (*AtCRP1p*, -1062 to -2 upstream the translation starting codon) was cloned into pBGWFS7 destination vector and introduced into Arabidopsis wild type background, ecotype Columbia-0 (Col-0), by *Agrobacterium tumefaciens*-mediated transformation. *At*CRP1-GFP transgenic lines were obtained by transformation of *AtCRP1*/*atcrp1-1* heterozygous plants with either the *At*CRP1 coding sequence fused to GFP under the control of *35S-CaMV* promoter, cloned into pB7FWG2 vector, or the genomic locus fused to GFP under the control of the native promoter, cloned into a modified pGreenII vector (Gregis et al., 2009). The GUN1 coding sequence, devoid of the stop codon, was cloned into pB7RWG2 vector, carrying an RFP reporter gene. pB7FWG2, pBGWFS7, and pB7RWG2 plasmids were obtained from Flanders Interuniversity Institute for Biotechnology of Gent (Karimi et al., 2002). Primers used for amplification of the DNA fragments cloned into the vectors, reported above, are listed in **Supplementary Table S2**. Arabidopsis Col-0 and mutant plants were grown on soil under controlled growth chamber conditions with a 16 h light/8 h dark cycle at 22°C/18°C. In the case of mesophyll protoplast preparation, Arabidopsis plants were also grown on soil in a growth chamber under the above reported conditions. Moreover, phenotypic characterization and molecular biology analyses were also conducted on plants grown on Murashige and Skoog (MS) medium (Duchefa)^3^, supplemented with or without 1% (w/v) sucrose. Tobacco plants, employed for transient gene expression, were cultivated for 5–6 weeks in a greenhouse under a 12 h light/12 h dark cycle at 22°C/18°C.

### Protoplast Transformation

Mesophyll protoplasts of *Arabidopsis thaliana* (Col-0) were isolated and transiently transformed according to Yoo et al. (2007) and Costa et al. (2012). Briefly, well-expanded rosette leaves from 3-to-5 week-old plants were cut into strips of 0.5–1 mm with a fresh razor blade. Leaf tissue was digested using an enzyme solution containing 1.25% cellulase Onozuka R-10 (Duchefa) and 0.3% Macerozyme R-10 (Duchefa) for 3 h at 23°C in the dark. The protoplast suspension was filtered through a 50 μm nylon mesh washed three times with W5 solution (154 mM NaCl, 125 mM CaCl_2_, 5 mM KCl, 2 mM MES, pH 5.7 adjusted with KOH) and used for PEG-mediated transformation. For each protoplast transformation 10 μg of a MidiPrep purified DNA (QIAGEN) plasmid harboring the *35S-CaMV*::*AtCRP1-GFP* cassette was used. Protoplasts were maintained for 16–24 h at 23°C in the dark, before performing epifluorescent microscopy.

### Transient Expression in *Nicotiana benthamiana* Leaves

Tobacco leaf infiltration was performed using *A. tumefaciens* strain GV3101/pMP90 carrying the specified constructs (see results for details) together with the p19-enhanced expression system (Voinnet et al., 2003), according to the method described by Waadt and Kudla (2008). The final OD_600_ for *A. tumefaciens* strains harboring *35S-CaMV*::*AtCRP1-GFP* and *35S-CaMV*::*GUN1-RFP* was 0.2 and 0.3, respectively. After infiltration, plants were incubated for 3–5 days under the conditions described above.

### Confocal Microscopy Analysis

Confocal Scanning Laser Microscopy analyses were performed using an inverted microscope, Leica DMIRE2, equipped with a Leica TCS SP2 laser scanning device (Leica). For the simultaneous detection of GFP and chlorophyll auto-fluorescence the cells were excited (Arabidopsis mesophyll protoplasts or tobacco leaf cells) with the 488 nm line of the Argon laser and the emissions were collected between 515/535 and 650/750 nm, respectively. For RFP detection the cells were excited at 561 nm from a He/Ne laser and the emission was collected between 575/625 nm. Image analyses were performed with Fiji^4^: an open-source platform for biological-image analysis (Schindelin et al., 2012).

### Nucleic Acid Analyses

Arabidopsis DNA was isolated according to Ihnatowicz et al. (2004). Isolation of total RNA from homozygous *atcrp1-1* plants at four-leaf rosette stage and RNA gel blot analyses were performed as described by Meurer et al. (2002), using 10 μg of total RNA for each sample. For the RNA slot blot hybridization experiments, one-fourth of the RNA purified from each immunoprecipitation pellet and one-tenth of the RNA purified from the corresponding supernatant were applied to a nylon membrane with a slot-blot manifold and hybridized to specific radiolabeled probes (see **Supplementary Table S2**). ^32^P-labeled DNA probes, complementary to chloroplast genes, were amplified using the primer pairs listed in **Supplementary Table S2**. Four micrograms of total RNA, treated with TURBO DNA-*free* (Ambion by Life Technologies), were employed for first-strand cDNA synthesis using GoScript Reverse Transcription System (Promega) according to the supplier’s instructions. Quantitative Real-Time PCR (qRT-PCR) was carried out on an CFX96 Real-Time system (Bio-Rad), using the primer pairs reported in **Supplementary Table S2**. The *SAND* (Remans et al., 2008) and *ubiquitin* transcripts were used as internal references. Data from three biological and three technical replicates were analyzed with Bio-Rad CFX Manager software (V3.1).

### Immunoblot Analyses

For immunoblot analyses, total proteins were prepared as described by Martinez-Garcia et al. (1999). Total proteins, corresponding to 5 mg of leaf fresh-weight (100% of WT and *atcrp1-1* samples) and isolated from plants at four-leaf rosette stage, were fractionated by SDS–PAGE (12% acrylamide [w/v]; (Schagger and von Jagow, 1987). Proteins were then transferred to polyvinylidene difluoride (PVDF) membranes (Ihnatowicz et al., 2004) and replicate filters were immunodecorated with antibodies specific for PSI (PsaA, PsaC, and PsaD), PSII (D1, PsbO) Cyt *b_6_*/*f* (PetA, PetB, and PetC), ATPase (ATPase-β) subunits, PSI (Lhca1, Lhca2) and PSII (Lhcb2, Lhcb3) antenna proteins, all obtained from Agrisera^5^. The GFP antibody was purchased from Life Technologies^6^.

### Chloroplast Stromal Preparation and Protein Immunoprecipitation

Intact chloroplasts were isolated from 11 days old Arabidopsis plants, according to Kunst (1998), and Kupsch et al. (2012) with some modifications. Chloroplasts were directly resuspended in 300–400 μl of extraction buffer [2 mM DTT, 30 mM HEPES-KOH, pH 8.0, 60 mM KOAc, 10 mM MgOAc and proteinase inhibitor cocktail (Sigma–Aldrich-P9599)]. Two independent stromal preparations were carried out and one of them was performed in the presence of 2% sodium deoxycholate in order to solubilize the membrane-attached *At*CRP1 protein fraction. Chloroplasts were then disrupted by pulling them through a syringe (0.55 mm × 40 mm) 30–40 times. The solution was centrifuged at 21,000 × *g* at 4°C to separate the stromal from the membrane fraction.

The isolated stromal fraction was diluted with one volume of coimmunoprecipitation (CoIP) buffer (150 mM NaCl, 20 mM Tris-HCl pH 7.5, 2 mM MgCl_2_, 0.5% Nonidet P-40 and 0.5 μg/mL Aprotinin). Five microliters of mouse anti-GFP antibody (Roche, No. 11814460001) were added to the stromal fraction and incubated for 1 h at 4°C and 13 rpm on an overhead shaker. Thereafter the coimmunoprecipitation was performed as described by Kupsch et al. (2012). Successful precipitation of *At*CRP1-GFP was confirmed by immunoblot analyses, using the same GFP antibody.

### RNA Extraction and Labeling for RIP-Chip Assay

RNA immunoprecipitation-chip analyses were performed using a tiling microarray covering the complete Arabidopsis chloroplast genome (Kupsch et al., 2012). The coimmunoprecipitated RNA was isolated from pellet and supernatant fractions either by phenol-chloroform extraction or using the Direct-zol^TM^ RNA MiniPrep kit (Zymo Research). For the phenol-chloroform extraction, RNA samples were incubated in 1% SDS and 5 mM EDTA at room temperature for 5 min to dissociate RNA-protein complexes. RNA was phenol-chloroform extracted, ethanol precipitated with the addition of Glycoblue^TM^ Coprecipitant (Thermo Fisher Scientific), washed twice with 75% ethanol, air-dried and resuspended in 20 μl RNase-free water. For the replicate, RNA was extracted using the Direct-zol^TM^ RNA MiniPrep kit (Zymo Research) according to the manufacturer’s instructions. Before the extraction 2 μg yeast RNA was added to the coimmunoprecipitated RNA pellet. The entire RNA of the pellet fraction and 2 μg RNA of the supernatant fraction were used for labeling. The pellet and supernatant RNA were labeled with 0.5 μl Cy5 and 1 μl Cy3 dye, respectively (aRNA labeling kit, Kreatech Diagnostics). Labeling reaction, microarray hybridization, scanning, and evaluation were performed as described in Kupsch et al. (2012). Only PCR products for which more than half of all replicate spots (24 per PCR product spanning two experiments) passed our quality assessment (Kupsch et al., 2012) and were used in this analysis (**Supplementary Table S1**).

### *In silico* Prediction of AtCRP1 Binding Sites

The putative *At*CRP1 binding motif, i.e., the nucleotide preference for each of the amino acid pairs at the fifth and last position of PPR domains, was predicted *in silico* using the reported weighting schemes (Barkan et al., 2012; Barkan and Small, 2014; Harrison et al., 2016). The software FIMO^7^, which analyzes sequence databases for occurrences of known motifs (Grant et al., 2011), was employed to identify the potential binding sites of *At*CRP1 within the regions enriched in our RIP-Chip experiment. Furthermore, the same regions were searched for the presence of sRNA native footprints, by consulting the JBrowse sRNA database^8^ (Ruwe et al., 2016). Numbers that delimit the native footprints refer to the chloroplast genome of *Arabidopsis thaliana* (NC_000932.1).

### β-Glucuronidase (GUS) Assay

For GUS histochemical detection, plant material was fixed in 90% acetone at -20°C for 1 h. Samples were then washed three times with NaPi buffer (NaH_2_PO_4_ 50 mM, Na_2_HPO_4_ 50 mM; pH 7.0) and stained overnight at 37°C with *X*-gluc solution [1 mM 5-bromo-4-chloro-3-indolyl-β-D-clucoronide, 2 mM K_3_/K_4_Fe(CN)_6_, 0,1% Triton (v/v), 10 mM EDTA, 50 mM NaPi pH 7.0]. 70% EtOH (v/v) was used as washing solution. Stained samples were then stored at 4°C and observed using a Zeiss Axiophot D1 microscope equipped with differential interference contrast (DIC) optics. Images were recorded with an Axiocam MRc5 camera (Zeiss) using the Axiovision program (v.4.1).

## Results

### *At*CRP1 Is a PPR Protein Imported into the Chloroplast

**FIGURE 1:**
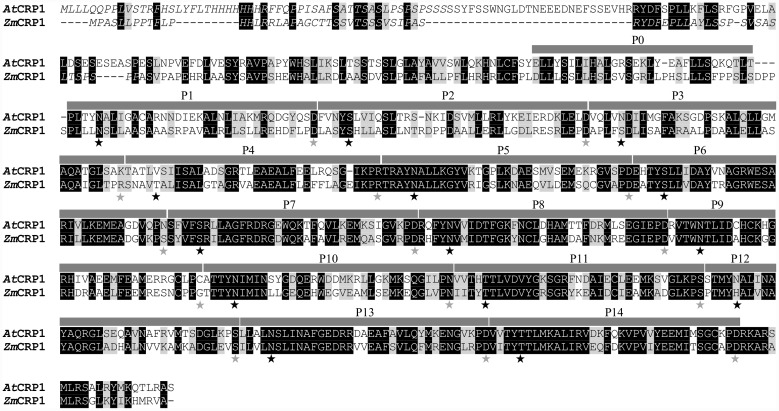
**Primary amino acid sequence alignment of *At*CRP1 and *Zm*CRP1 proteins.** The amino acid sequence of the Arabidopsis CRP1 (*At*CRP1, At5g42310) was compared with CRP1 from *Zea mays* (*Zm*CRP1), using ClustalW2. Black boxes indicate strictly conserved amino acids, and gray boxes closely related ones. The predicted chloroplast transit peptides (ChloroP, http://www.cbs.dtu.dk/services/ChloroP/) are indicated in italics, and the PPR motives (P0-to-P14), identified using the PlantPPR database (http://www.plantppr.com), are marked with gray bars. The specificity determining amino acids in each PPR motif at position 5 and 35 are indicated by black and gray stars, respectively. Note that P0 motif was not considered to contribute to the identification of RNA targets, as previously reported by Barkan et al. (2012). P0 is composed of 30 aa, whereas all other P motifs are of 35 aa, with the exception of P2, wich contains 37 aa in Arabidopsis and 38 in maize.

The Maize Genetics and Genomics Database (Lawrence et al., 2004)^9^ was used to identify the At5g42310 gene as the Arabidopsis ortholog of *ZmCRP1* (see also Belcher et al., 2015). At5g42310 encodes a polypeptide of 709 amino acids with a calculated molecular mass of 80 kDa. Intron number (three) and position are conserved between the two genes, and BLASTP query of public Arabidopsis sequence database with *Zm*CRP1 amino acid sequence detected At5g42310 protein as the top hit with 55% sequence identity and 72% sequence similarity (**Figure 1**).

**FIGURE 2:**
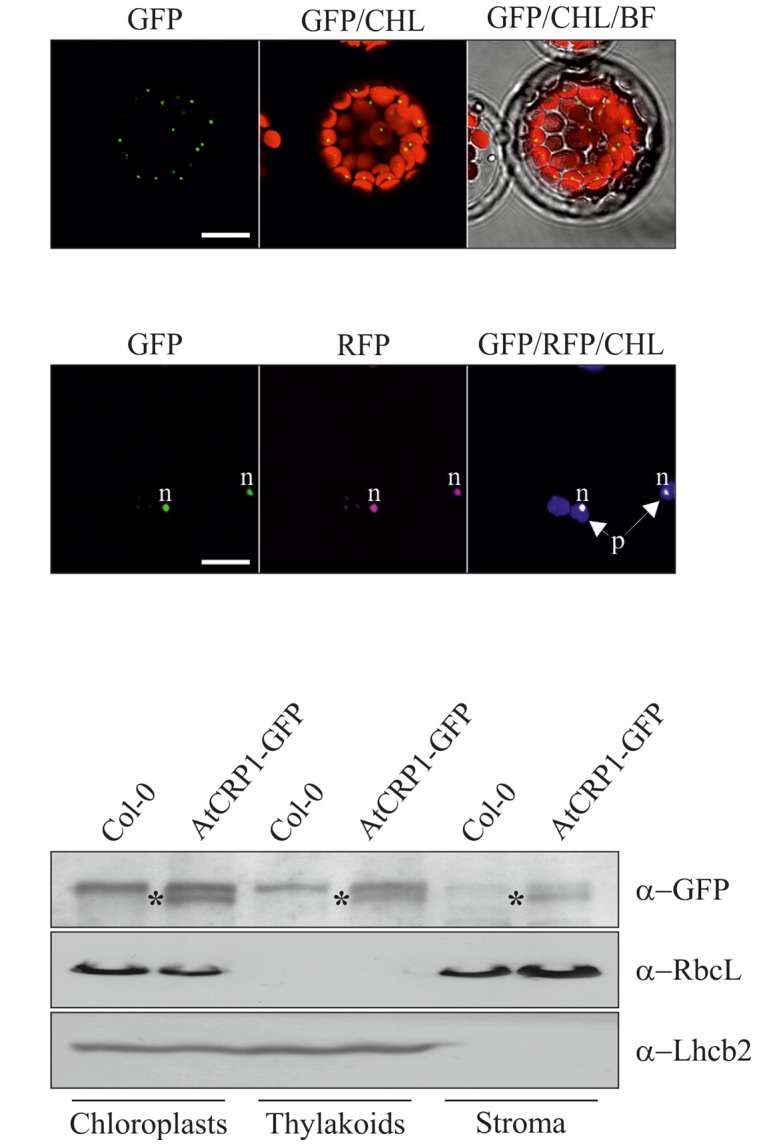
**Subcellular localization of *At*CRP1 in Arabidopsis mesophyll protoplasts and leaf cells.** **(A)** Series of Lasers Scanning Confocal images (CLSM) of the subcellular localization of the *At*CRP1-GFP fusion protein (indicated as GFP) expressed in transiently transformed Arabidopsis (ecotype Col-0) leaf mesophyll protoplasts. The GFP signal accumulates in distinct spots within the chloroplasts, visualized by the red chlorophyll autofluorescence (CHL), resembling the pattern of chloroplast nucleoids. BF, Bright Field. **(B)** Series of CLSM images of the subcellular localization of *At*CRP1-GFP and GUN1-RFP [indicated as RFP and used as a marker of chloroplast nucleoids (Koussevitzky et al., 2007)] fusion proteins upon transient co-expression in tobacco leaf cells. The green fluorescence (GFP) co-localizes perfectly with the purple fluorescence (RFP) inside the chloroplasts (violet autofluorescence of chlorophylls, CHL), indicating that *At*CRP1 protein is part of the chloroplast nucleoids. Images are representative of three independent experiments. Bar = 10 μm; *p* = chloroplast; *n* = nucleoid. **(C)** Immunoblot analyses of proteins from Col-0 and Arabidopsis transgenic lines containing the *AtCRP1-GFP* construct under the control of *AtCRP1* native promoter (approximately 1 kb upstream of the translation start codon, see also Materials and Methods). Equal protein amounts isolated from total chloroplasts, thylakoids and stroma were loaded. Filters were immunolabeled with a GFP specific antibody to detect the localization of the *At*CRP1-GFP chimera. An antibody specific for the large subunit of RUBISCO (RbcL) was used as a marker of chloroplast stroma, whilst an Lhcb2 specific antibody was used as a marker of thylakoid membranes. Asterisks indicate the position of the AtCRP1-GFP fusion protein. One out of three immunoblots for each antibody is shown. Note that the *At*CRP1-GFP chimera is fully functional, since it was able to rescue the *atcrp1-1* mutant phenotype (see also **Figure 4**).

*At*CRP1 is annotated as a PPR protein and shares with *Zm*CRP1 15 PPR tandem repeats, which were predicted by using the PlantPPR database (Cheng et al., 2016). All PPR motifs are of 35 aa, with the exception of P0 which consists of 30 aa and P2 of 37 aa in Arabidopsis and 38 aa in maize. The fifth and the last residue of each PPR domain form the amino acid pairs that specify the RNA target molecules (Cheng et al., 2016), and are labeled with gray and black stars in **Figure 1**. The ChloroP server (Emanuelsson et al., 1999)^10^ predicted the presence of a cTP of 54 residues (see amino acid residues in italics in **Figure 1**), indicating that *At*CRP1, like *Zm*CRP1, could be imported into the chloroplast. To corroborate the *in silico* prediction, the *At*CRP1-GFP fusion protein was expressed in transiently transformed Arabidopsis protoplasts (**Figure 2**). In agreement with the ChloroP prediction, the chimeric protein (GFP fluorescence) accumulated within the chloroplast in distinct fluorescent foci (CHL, autofluorescence of chloroplast chlorophylls, **Figure 2A**), resembling the nucleoid complexes. Indeed, *At*CRP1-GFP chimera co-localized perfectly with the GUN1-RFP fusion protein, used as a nucleoid marker in this assay (RFP fluorescence, **Figure 2B**), (Koussevitzky et al., 2007; Colombo et al., 2016; Tadini et al., 2016), in tobacco leaf cells. To further localize *At*CRP1, chloroplasts were fractionated to separate the stroma and thylakoid compartments. Immunoblot analysis, using a GFP specific antibody, allowed detection of *At*CRP1-GFP specific signal in total chloroplasts, as well as in thylakoids and in the stromal fraction, indicating that the nucleoid *At*CRP1 protein is both associated to membranes and soluble in the stroma (**Figure 2C**). These findings are in agreement with the identification of *At*CRP1 as part of Megadalton complexes in the chloroplast stroma (Olinares et al., 2010), as well as in the grana of thylakoid membranes (Tomizioli et al., 2014).

### *At*CRP1 Is Essential for Plant Autotrophy

**FIGURE 3:**
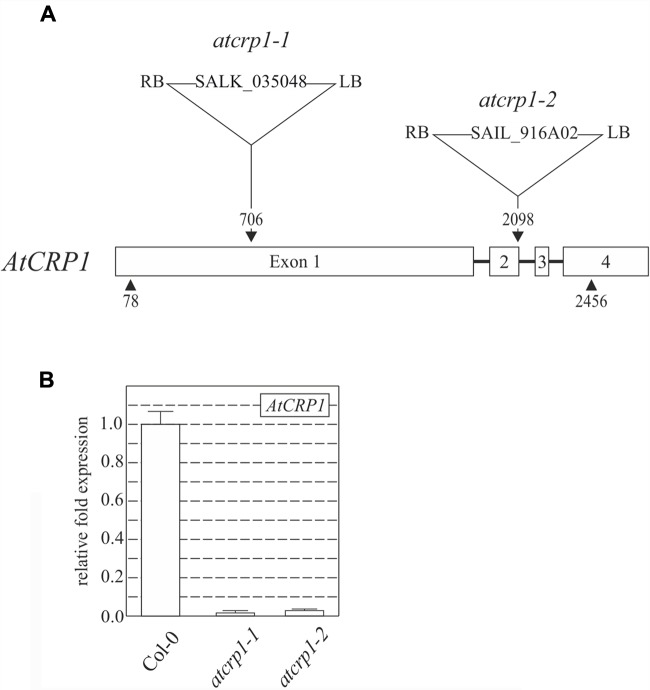
**T-DNA tagging and expression levels of *AtCRP1* gene.** **(A)** Schematic representation of *AtCRP1* gene, where exons are indicated as numbered white boxes, while introns are shown as black lines. Arrowheads indicate the positions of translation initiation and stop codons. The locations, designations and orientations of T-DNA insertions are indicated (RB, right border; LB, left border). Note that the T-DNA insertions are not drawn to scale. **(B)** Levels of *AtCRP1* gene expression was ascertained by real-time PCR of cDNA obtained from leaves of WT (Col-0) and *atcrp1-1. atcrp1-2* mutant plants. Gene expression was normalized to the level of *AtCRP1* transcripts in Col-0 plants, and *SAND* and *ubiquitin* were used as internal references. The bars indicate standard deviations.

To investigate the role that *At*CRP1 plays in Arabidopsis, two lines carrying T-DNA insertions into the coding sequence of At5g42310, renamed *atcrp1-1* (Salk_035048) and *atcrp1-2* (Sail_916A02), were obtained from the T-DNA Express Arabidopsis mutant collection (**Figure 3A**; see also Materials and Methods).

**FIGURE 4:**
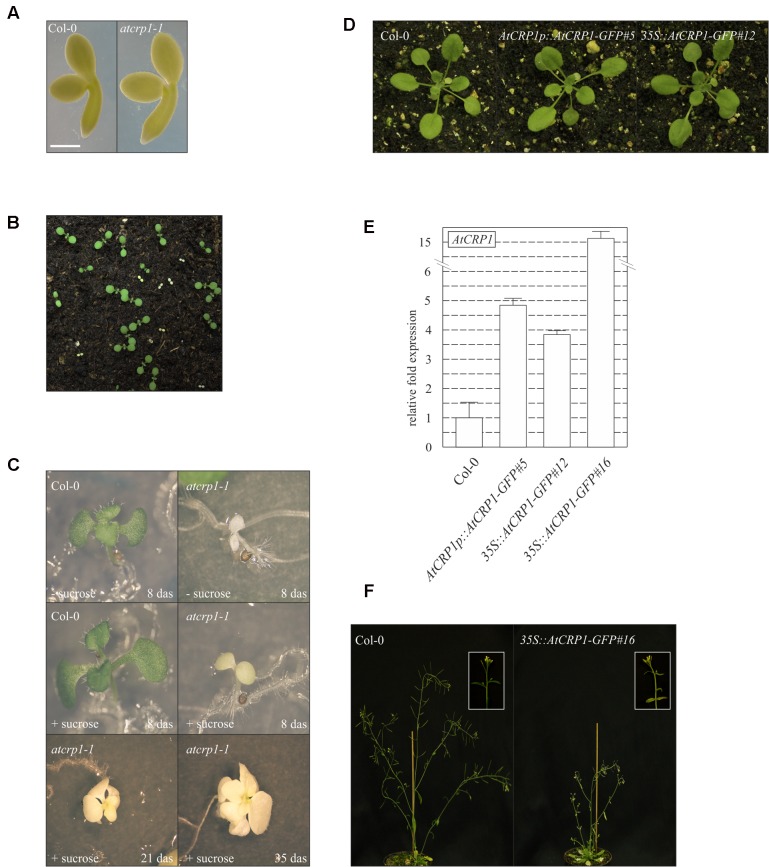
**Effects of loss of *At*CRP1 on plant development.** **(A)** Images of isolated fully mature embryos (bent cotyledon stage) from WT (Col-0) and *atcrp1-1* seeds. The lack of *At*CRP1 protein did not alter embryo development, although mutant embryos were slight larger and paler than Col-0. **(B)** *atcrp1-1* seeds were able to germinate on soil, giving rise to yellow seedlings that accounted for about one-quarter of all seedlings, indicative of a monogenic recessive trait. Mutant seedlings did not survive past the cotyledon stage. **(C)** Mutant seedlings showed albino cotyledons when grown on MS medium without sucrose and arrested at the cotyledon stage as in **(B)**. However, when *atcrp1-1* seedlings were grown on MS medium supplement with 1% sucrose, they showed yellow-albinotic cotyledons at 8 das (days after sowing) and were able to develop up to 8–10 true leaves after 35 das. **(D)** The *atcrp1-1* seedling lethal phenotype could be fully rescued by *Agrobacterium tumefaciens*-mediated transformation of *AtCRP1*/*atcrp1-1* heterozygous plants with either the *AtCRP1* coding sequence fused to GFP under the control of 35S-CaMV promoter (*35S::AtCRP1-GFP#12*), or the genomic sequence fused to GFP under the control of native promoter (*AtCRP1p::AtCRP1-GFP#5*). **(E)** Real-time PCR to monitor the expression of *AtCRP1* gene in WT and complemented plants. Gene expression was normalized with respect to the level of *AtCRP1* transcripts in Col-0, and *SAND* and *ubiquitin* were used as internal references. The bars indicate standard deviations. **(F)** Col-0 and *35S*::*AtCRP1-GFP#16* transgenic line with about 15-folds more *AtCRP1* transcripts than WT. In this case the transgenic line shows WT-like rosette, but it is characterized by shorter and paler stems, with bleached cauline leaves and sterile flowers. A detail of the stem and inflorescence is shown in the inset. Note that the detailed molecular characterization of *At*CRP1 function was conducted on *atcrp1-1* plants, since the *atcrp1-2* seedlings showed an identical phenotype.

Both T-DNA insertions completely suppressed the accumulation of the corresponding transcripts in homozygous mutant seedlings (**Figure 3B**), which were characterized by a paler pigmentation of cotyledons, visible even at the fully mature embryo stage (**Figure 4A**), and leaves (**Figures 4B,C**), and found to be seedling lethal under autotrophic growth conditions on soil and MS medium without sucrose, but able to develop yellow-albinotic rosette leaves and sterile inflorescence when sucrose was provided in the medium (**Figure 4C**). The mutant phenotype could be rescued by *Agrobacterium tumefaciens*-mediated transformation of heterozygous plants with either the appropriate coding sequence fused to the 35S promoter of cauliflower mosaic virus (*35S-CaMV*::*AtCRP1-GFP*), or the genomic sequence including a 1-Kbp fragment of the promoter region (*AtCRP1p*::*AtCRP1-GFP*), corroborating a direct correspondence between genotype and phenotype, and indicating that the AtCRP1-GFP chimera was fully functional, in both cases (**Figure 4D**). Interestingly, complemented plants carrying the *AtCRP1-GFP* construct under the control of the native promoter showed a fivefold increase in *AtCRP1* gene expression (**Figure 4E**), most probably as consequence of the T-DNA insertion in a highly expressed euchromatin region of the nuclear genome. Furthermore, a complete rescue of mutant plant phenotype could only be observed in *35S*::*AtCRP1-GFP* transgenic lines with a limited accumulation of *AtCRP1* transcripts (**Figures 4D,E**). Higher *AtCRP1* expression levels (around 15-folds in comparison to WT) led to transgenic plants with WT-like rosette but shorter and paler stems, bleached cauline leaves, together with sterile flowers (**Figures 4E,F**).

**FIGURE 5:**
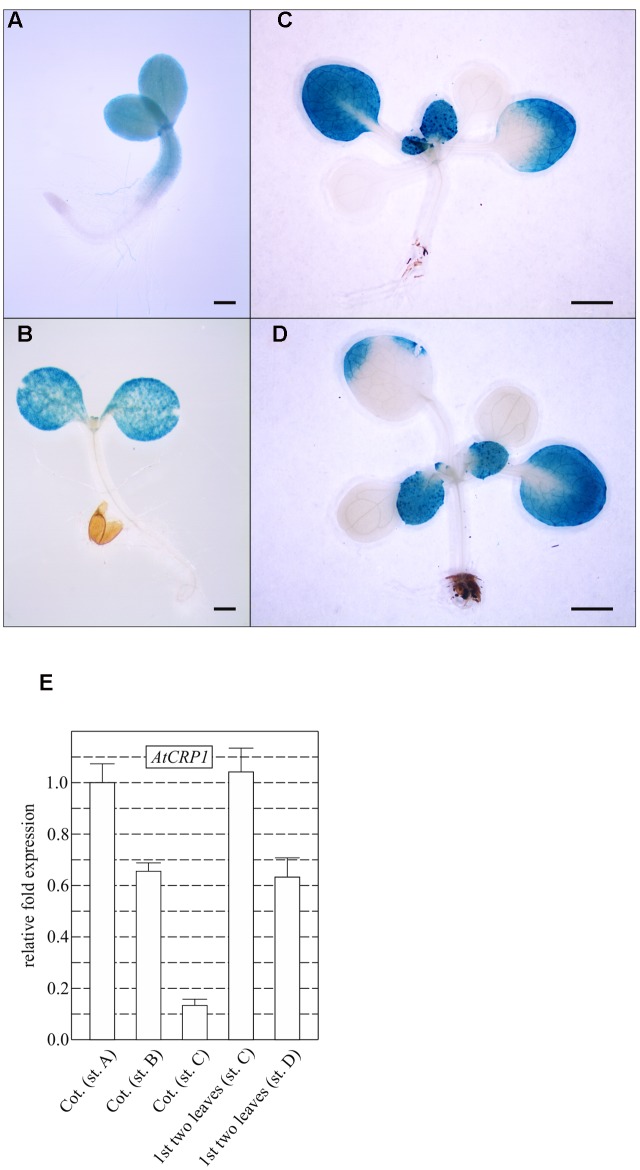
***AtCRP1* promoter-driven β-glucuronidase (GUS) activity in cotyledons and rosette leaves.** Histochemical GUS staining was conducted on seedlings at the two cotyledon stage **(A)**, at the onset of the first true leaves **(B)**, at four leaves rosette-stage **(C)**, and at the onset of the third pair of true leaves **(D)**. In general, GUS staining in younger leaves was stronger than in older leaves and the activity of *AtCRP1* promoter was below the limit of detection in cotyledons after the development of the first true leaves. **(A)** and **(B)** Bar = 1 mm, **(C)** and **(D)** bar = 1 cm. **(E)** Real-time PCR analyses were conducted with cDNA obtained from cotyledons at the developmental stages reported in **(A–C)** (Cot. st. A, Cot. st. B and Cot. st. C) and on the first pair of true leaves at stages C–D (st. C and st. D) to monitor the accumulation of *AtCRP1* transcripts. Gene expression was normalized with respect to the level of *AtCRP1* transcripts in cotyledons at stage A, and *SAND* and *ubiquitin* were used as internal references. The bars indicate standard deviations.

Temporal and spatial expression patterns of *At*CRP1, monitored by fusing the promoter region of the gene upstream of the GUS reporter gene (see also Materials and Methods), support further the key role played by *At*CRP1 during early stages of seedling and leaf development (**Figure 5**). The GUS staining could, indeed, be detected in young cotyledons and in the upper portion of the hypocotyl (**Figure 5A**). Furthermore, intense GUS signals were observable in young developing leaves (**Figures 5C,D**), whereas the GUS coloration tended to decrease in old cotyledons and leaves (**Figures 5B–D**). Similar results were also obtained by monitoring the expression of *AtCRP1* in cotyledons and leaves using quantitative Real-Time PCR (qRT-PCR). In general, a high level of expression of *AtCRP1* was observed in green developing tissues, such as young cotyledons and leaves, whereas the expression decreased in older tissues (**Figure 5E**).

### *atcrp1* Mutant Chloroplasts Fail to Accumulate Cytochrome *b_6_/f* Protein Complex and the PsaC Subunit of PSI

**FIGURE 6:**
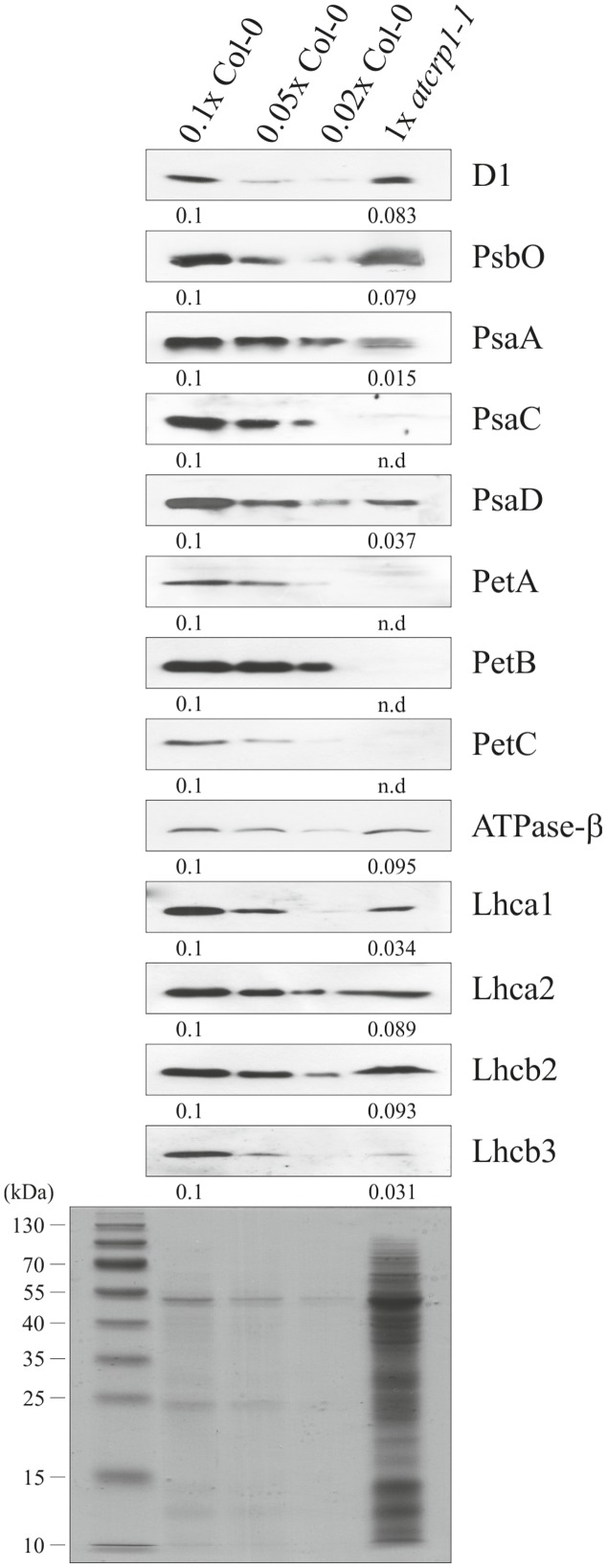
**Immunoblot analyses of thylakoid protein complexes in Col-0 and *atcrp1-1* mutant leaves.** PVDF filters bearing fractionated total proteins, isolated at the four-leaf rosette stage from Col-0 and *atcrp1-1* plants grown on MS medium supplemented with 1% sucrose (see also **Figure 4**), were probed with antibodies raised against individual subunits of PSII (D1, PsbO), PSI (PsaA, PsaC, and PsaD), Cyt *b_6_f* (PetA, PetB, and PetC), ATPase (ATPase-β), LHCI (Lhca1, Lhca2) and LHCII (Lhcb2, Lhcb3). Reduced levels of Col-0 total proteins were loaded in the lanes marked 0.1x Col-0, 0.05x Col-0, and 0.02x Col-0 in order to obtain signals from Col-0 proteins within the range of mutant protein signals (1x *atcrp1-1*). A replica SDS-PAGE stained with Coomassie-brilliant-blue is shown as loading control. Averaged relative protein abundance is given below each immunoblot and standard deviation was less than 10%. One out of three immunoblots for each antibody is shown. Note that the complete lack of Cyt *b_6_f* and PsaC subunits was also observed in *atcrp1-2* leaves. n.d., not detected.

The albino pigmentation of *atcrp1* seedlings, together with their inability to grow under autotrophic conditions, indicated a defect in the thylakoid-associated photosynthetic apparatus. To verify this assumption, immunoblot analyses with antibodies specific for single subunits of the four major thylakoid protein complexes were performed on total leaf proteins. Leaf samples were harvested from *atcrp1* plants at the four-leaf rosette stage and grown on MS-medium supplemented with 1% sucrose (**Figure 6**; see also Materials and Methods). Under standard light conditions (50 μmol photons m^-2^ s^-1^), subunits of Photosystem I (PsaA, PsaC, and PsaD), Photosystem II (D1, PsbO), Light harvesting complexes (Lhca1, Lhca2, Lhcb2, and Lhcb3) and ATPase (ATPase-β) accumulated to levels lower than 10% with respect to wild type plants. Furthermore, subunits of the Cyt *b_6_/f* (PetA, PetB, and PetC) and PSI (PsaC) were below the limits of immunoblot detection.

In summary, these results indicate a general reduction of thylakoid protein complex subunits in *atcrp1* leaves, with a particularly severe effect on the accumulation of the Cyt *b_6_/f* complex and PsaC.

### *AtCRP1* Is Associated *In vivo* with *psaC* and *petB-petD* Transcripts

**FIGURE 7:**
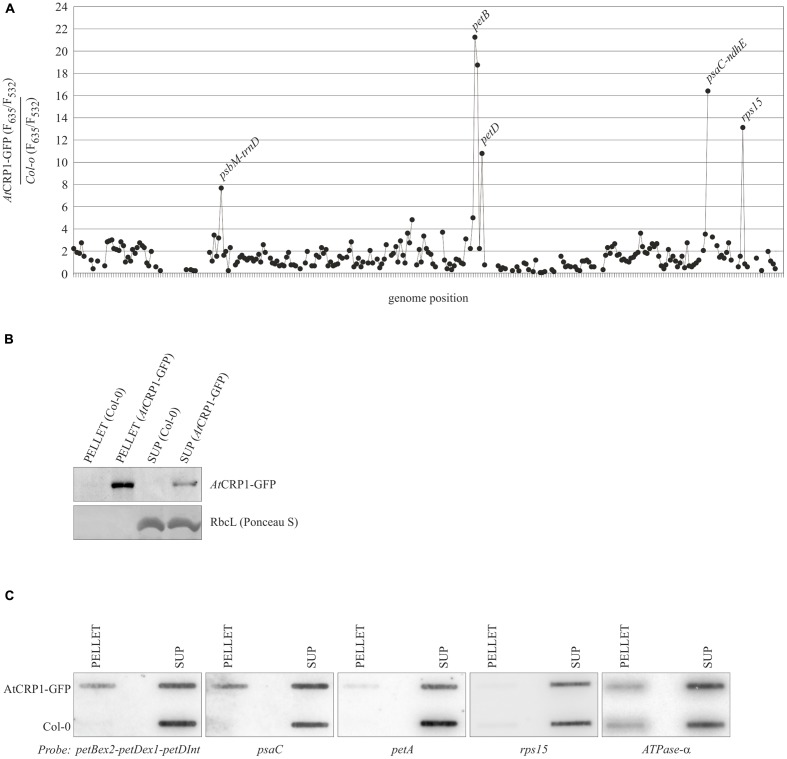
***At*CRP1 RIP-Chip data plotted according to gene order within the plastid genome.** **(A)** Differential enrichment ratios obtained by RNA immunoprecipitation (RIP)-Chip analysis. The enrichment ratios (*F*_635_/*F*_532_) obtained from an assay of *AtCRP1p::AtCRP1-GFP* chloroplast stroma extract were normalized with respect to a control assay that used WT (Col-0) chloroplast stroma extract (both assays were performed in duplicate). The median-normalized values for replicate spots were plotted according to gene order within the plastid genome. Fragments for which fewer than 13 spots per experiment (*AtCRP1p::AtCRP1-GFP*/WT) passed our manual quality control and/or yielded an F532 signal below background were excluded and appear as gap in the curve. The enrichment of *psaC* 5′UTR is in agreement with previous findings obtained by RIP-Chip analysis on CRP1 from maize (Schmitz-Linneweber et al., 2005). **(B)** Immunoblot analysis of protein fractions obtained from immunoprecipitation experiments using the anti-GFP mouse antibody and stroma material from Col-0 and *At*CRP1-GFP plants. Equal volumes of supernatant and pellet preparations were loaded onto the gel. Note that the pellet from *At*CRP1-GFP immunoprecipitation gave a stronger signal than the corresponding supernatant, implying quantitative precipitation of *At*CRP1-GFP. The fact that no signal was obtained with Col-0 extracts demonstrates the specificity of the antibody. The RbcL migration region of the Ponceau S stained nylon membrane, after transfer from SDS-PAGE, was used to verify equal loading. **(C)** Verification of *At*CRP1 RNA targets. Coimmunoprecipitations and RNA extractions from *At*CRP1-GFP and Col-0 samples were performed as for RIP-Chip assays. The RNAs were then analyzed by slot-blot hybridization with the indicated probes (see also Materials and Methods and **Supplementary Table S2**). The *ATPase-α* probe hybridization was included as a control. SUP, supernatant.

*Zm*CRP1 has been previously demonstrated to associate with the *psaC* and *petA* mRNAs *in vivo* by RIP-Chip analyses (Schmitz-Linneweber et al., 2005). To investigate whether *At*CRP1 shares with *Zm*CRP1 the RNA targets, the same RIP-Chip approach employed in maize was used here. Stroma from plants expressing *At*CRP1-GFP, under the control of the native promoter (*AtCRP1p*::*AtCRP1-GFP*), was isolated and the fusion protein was immunoprecipitated using an anti-GFP serum. As a control, we performed mock precipitations with stroma extracted from WT plants, using the same GFP antibody. RNA was purified from the immunoprecipitation pellets and supernatants and was labeled with Cy5 (red) and Cy3 (green) fluorescent dyes, respectively. The two RNA fractions from *At*CRP1-GFP immunoprecipitations (IPs) and from mock IPs were competitively hybridized to a chloroplast genome tiling microarray (Kupsch et al., 2012). Enrichment of RNA is reflected in the ratio of red to green fluorescence for each spot on the microarray. Two biological replicate experiments were performed with stroma from *At*CRP1-GFP expressing plants and two with WT stroma. Data from the four assays were normalized and used to calculate median enrichment ratios of the red and green fluorescence signals for each PCR product among the 24 replicate spots on two arrays (**Supplementary Table S1**). To identify enrichment of RNA species specifically in the *At*CRP1-GFP immunoprecipitation, we plotted the difference in median enrichment ratio for each DNA fragment between the *At*CRP1-GFP and mock experiment against the position of the product on the plastid chromosome (**Figures 7A,B**).

Four prominent peaks of differential enrichment were observed. One of them corresponds to the 5′UTR of *psaC* transcript, a target already recognized as a ligand of *Zm*CRP1 in RIP-Chip assays (Schmitz-Linneweber et al., 2005). A second RNA target is represented by the *petB-petD* intergenic region. This RNA was not identified to interact with *Zm*CRP1 by RIP-Chip analysis, however, *Zm*CRP1 is known to aid in maturation of this particular intergenic region (Barkan et al., 1994). Interestingly, the observed enrichment of *rps15* transcripts might uncover a further, novel target of *At*CRP1, whereas the enrichment of *psbM/trnD* transcripts is often observed in RIP-Chip experiments, thus this peak was considered an artifact.

To corroborate the RIP-Chip data, the *At*CRP1-associated RNAs were analyzed by slot blots (**Figure 7C**). RNA purified from immunoprecipitation pellets and supernatants were probed with the PCR fragments that detected the most highly enriched sequences in the RIP-Chip assay. The data confirmed that the *psaC* and *petB-petD* transcripts are highly enriched in the *At*CRP1-GFP immunoprecipitates, but not the *rps15* RNA. *Zm*CRP1 was also reported to be associated with RNAs of the *petA* region (Schmitz-Linneweber et al., 2005; Williams-Carrier et al., 2008), however, no enrichment of *petA* transcripts could be observed in the *At*CRP1-GFP RIP-Chip assay (**Figure 7A**) and a low enrichment was detected in the slot blot assay (**Figure 7C**), possibly indicating that the interaction of *At*CRP1 with *petA* transcripts is not very stable. In general, our analysis cannot exclude the possibility that CRP1 binds to additional target RNAs, for example when interactions take place at chloroplast membranes. Since we are not using cross-linked material, weak RNA-protein interactions might be lost during our assay.

**FIGURE 8:**
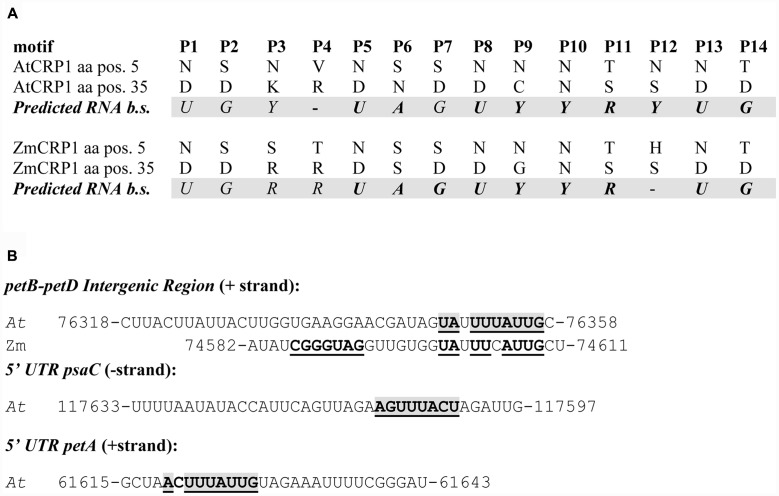
***At*CRP1 RNA binding sites and the chloroplast *in vivo* footprints.** **(A)** PPR motifs in *At*CRP1 were identified with the aid of PlantPPR database [www.plantppr.com, (Cheng et al., 2016). Amino acid residues in the 5th and last position of PPR motifs have been considered critical for sequence-specific RNA recognition, as previously reported (Barkan et al., 2012; Barkan and Small, 2014; Cheng et al., 2016; Harrison et al., 2016); see also **Figure 1**]. When the code developed for the different amino acid pairs is applied to the *At*CRP1 repeats, the sequence UGYNUAGUYYRYUG emerges as predicted RNA binding sequence (b.s.), whereas the sequence UGRRUAGUYYRNUG is predicted for *Zm*CRP1, in agreement with Barkan et al. (2012). **(B)** The sequences of *in vivo* footprints identified in the *petB-petD* intergenic region (Arabidopsis and Maize) and 5′UTR *psaC* region that co-map with *At*CRP1 binding sites (*p*-value < 0.01, highlighted in bold on a gray background) are shown. The Arabidopsis 5′UTR of *petA* transcripts shows also the presence of a native footprint that co-maps with *At*CRP1 binding site, however, this region was only enriched in the slot blot, but not in the AtCRP1 RIP-Chip assay (see **Figure 6**). There is no published sRNA within the *psaC* or *petA* 5′UTR of maize. A predicted binding site for maize CRP1 in the 5′-UTR of *psaC* (UGGAUAAACCAUUG; Barkan et al., 2012) is not similar in sequence to the Arabidopsis prediction shown here. Moreover, nucleic acid binding assay showed a direct interaction of *Zm*CRP1 with the 5′-UTR of *petA* (UUAGCUACCUAUCU**A**A**UUUAUUG**UAGAAAUU; Williams-Carrier et al., 2008), that shows high similarity with the corresponding Arabidopsis sequence (see predicted binding site highlighted in bold). Note that no *At*CRP1-specific *in vivo* footprint could be identified in the other RIP-Chip enriched regions, *rps15* and *psbM* (see also **Figure 7**).

To support further the RIP-Chip findings, *At*CRP1 target RNAs were interrogated for the presence of native footprints at the JBrowse database^11^. The JBrowse database provides annotations of *Arabidopsis thaliana* organellar short RNA (sRNA), thought to be generated from protein-mediated temporary protection of target RNAs against exonucleolytic degradation (Ruwe et al., 2016; see also **Figure 8**). sRNAs were found within the 5′UTR of *psaC* (corresponding to the 117633–117597 region of chloroplast genome) and the *petB-petD* intergenic region (region 76318–76358), and an sRNA was also annotated in the 5′UTR of *petA* (region 61615–61643). Furthermore, *At*CRP1 predicted RNA binding motifs were shown to co-map with the native footprints, when the corresponding sequences were searched for the occurrence of the consensus binding motif with the FIMO program in the MEME suite^12^ (**Figure 8B**; Takenaka et al., 2013). A short RNA has been also mapped upstream of *rps15*, but this region was not enriched in the RIP-Chip assay and the match with the predicted binding site of *At*CRP1 is weaker than for the *psaC. petB-petD*, and *petA* sRNAs.

In summary, the RIP-Chip and slot blot data together with the colocalization of native footprints and *At*CRP1 RNA binding motifs indicate that *At*CRP1 likely binds directly to the 5′UTR of *psaC* and the *petB-petD* intergenic region and possibly to the 5′UTR of *petA*. On the contrary, the absence of an *At*CRP1-specific footprint within the *rps15* RNA, together with the failure of slot blot enrichment, makes any *At*CRP1-*rps15* interaction unlikely.

### *At*CRP1 Is Required for the Correct Processing of *psbB-psbT-psbH- petB-petD* Transcripts

**FIGURE 9:**
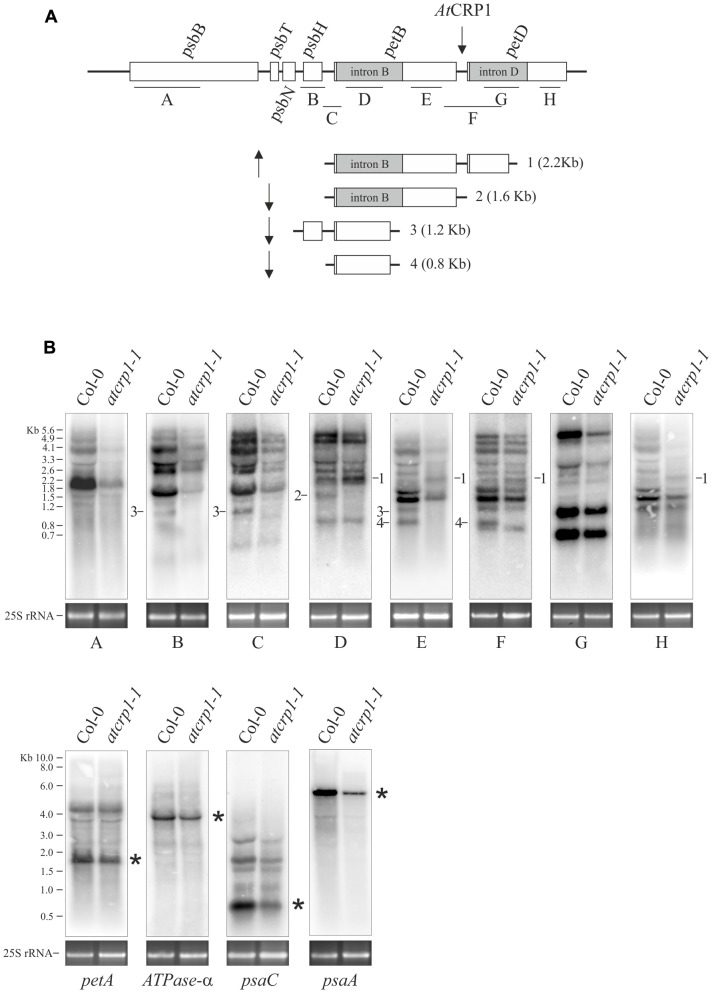
**Transcript patterns of chloroplast genes in Col-0 and *atcrp1-1* mutant leaves.** **(A)** The structure of the *psbB* gene cluster and probes A to H used in RNA gel blots analysis in **(B)** are shown. Furthermore, processed and spliced transcripts that accumulate differentially between Col-0 and mutant chloroplasts are drawn to scale and numbered from 1 to 4. Upward arrow indicates transcripts that accumulate to higher levels in *atcrp1-1* than Col-0 chloroplasts, whilst the downward arrow is used for transcripts less abundant or absent in mutant samples. The putative binding site of *At*CRP1 within the *petB-petD* intergenic region is also indicated. **(B)** RNA gel blot analysis of the *psbB* gene cluster were performed using probes indicated as A to H, whilst *petA. ATPase-A. psaC*, and *psaA* specific probes are described in section “Materials and Methods.” The identity of labeled transcripts (1–4), shown in **(A)** together with their size, was established based on the hybridization pattern, transcript size and on data reported in Meierhoff et al. (2003) and Stoppel et al. (2011). Asterisks indicate the mature transcript forms. A portion of the ethidium bromide stained Agarose gels, containing the cytosolic 25S rRNA, is included, as loading control, below each filter. One out of three Northern-blots for each transcript-specific probe is shown.

To assess whether the lack of Cyt *b_6_/f* complex and PsaC subunit, together with the marked reduction of all protein complex subunits observed in *atcrp1-1* thylakoids, was caused by deficiencies in transcript accumulation and *At*CRP1-dependent transcript processing, we probed the identified *At*CRP1 RNA targets and other plastid transcripts by gel blot hybridization (**Figure 9**).

We investigated the transcripts encoding the subunits CP47 (*psbB*), T (*psbT*), and H (*psbH*) of photosystem II (PSII), subunits A (*psaA*) and C (*psaC*) of PSI, Cyt *f* (*petA*), Cyt *b_6_* (*petB*) and subunit IV (*petD*) of cytochrome *b_6_/f* and the alpha subunit of ATPase (*ATPase-α*). All these transcripts accumulated in *atcrp1-1* plastids to levels lower than WT, indicating that global plastid gene expression is affected by the *atcrp1-1* mutation, and explaining the marked reduction of thylakoid protein accumulation observed in *atcrp1-1* leaves.

Furthermore, the plastid polycistronic transcription unit *psbB-psbT-psbH-petB-petD* showed some striking alteration of transcript pattern in *atcrp1* samples (**Figure 9**). In particular, the monocistronic *petB* (band #4, 0.8 Kb), the dicistronic *psbH-petB* (band #3; 1.2 Kb) and the unspliced *petB* (band #2, 1.6 Kb) transcripts were barely detectable in the mutant, whereas the *petB*-unspliced *petD*-spliced dicistronic transcript (band #1, 2.2 Kb), detected with probes D, E, F, and H, accumulated to even higher levels in *atcrp1* plastids, presumably due to the failure of *At*CRP1-dependent processing between the *petB* and *petD* coding regions, as also shown in *zmcrp1* mutant plants (Barkan et al., 1994; Fisk et al., 1999). In contrast with maize, monocistronic and spliced *petD* transcripts of ∼600 nucleotides do not accumulate to significant levels in Arabidopsis, and thus its absence was not observed in *atcrp1* plastids (Barkan et al., 1994; Barkan, 2011).

**FIGURE 10:**
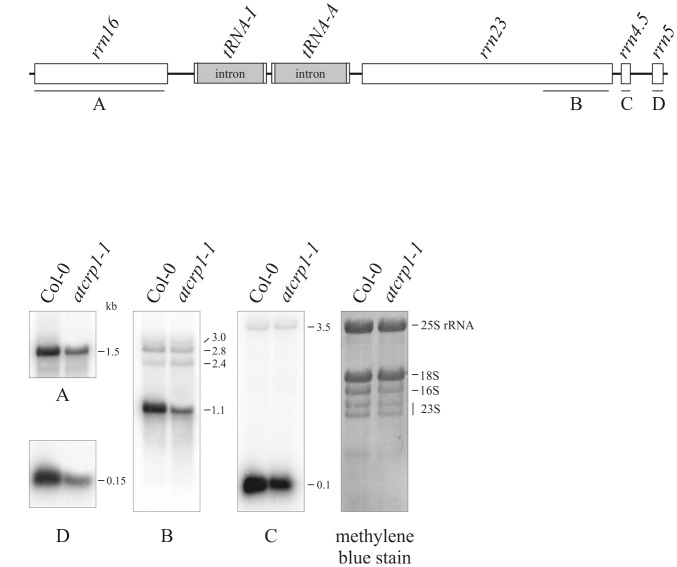
**Plastid rRNA accumulation in Colo-0 and *atcrp1-1* mutant leaves.** **(A)** Schematic representation of the chloroplast *rrn* operon. Probes used in Northern blot analysis are indicated as black bars under each rRNA gene (A–D). **(B)** RNA gel blot analysis of plastid rRNAs were performed using the probes A-to-D described above. For loading control, a methylene blue stained filter is shown. One out of three Northern-blots for each transcript-specific probe is shown.

Moreover, the lack of the PsaC and PetA subunits could be the consequence of the simultaneous decrease of transcript accumulation and a possible defect in *At*CRP1-dependent activation of *psaC* and *petA* translation, as shown in *Zea mays* (Barkan et al., 1994; Schmitz-Linneweber et al., 2005). However, the specific regulatory role of *AtCRP1* in plastid protein translation is difficult to verify, owing to the general and pleiotropic decrease of mature plastid *rRNA* in *atcrp1-1* leaves, in spite of WT-like accumulation of *rrn23* and *rrn4.5* precursor forms (**Figure 10**). This rRNA accumulation pattern is very similar to the ones of mutants with impaired chloroplast translation and has been interpreted as a secondary consequence of reduced plastid protein synthesis (Tiller et al., 2012; Tadini et al., 2016).

## Discussion

In this study we have investigated the role of *At*CRP1 in the biogenesis of dicotyledonous-C3 chloroplasts and compared its function to the already characterized monocotyledonous-C4 chloroplast counterpart, *Zm*CRP1. Both proteins are essential for chloroplast biogenesis and photosynthetic activity, since they are required for the processing and translation of specific plastid transcripts encoding subunits of the thylakoid protein complexes. Our results indicate that *At*CRP1 and *Zm*CRP1 have very similar RNA targets and the main functional divergences are most likely due to the distinct localization of the two proteins inside the chloroplast and the partially different affinity for the RNA targets (see **Table 1**).

**Table 1:** Overview of the phenotypes of Arabidopsis and maize *crp1* mutants and comparison of their molecular roles in chloroplast biogenesis.

*atcrp1^a^*							*zmcrp1^b^*					
**Plant phenotype**								
Seedling lethal with yellow-albinotic cotyledons and leaves. Plants are able to develop mature leaves and sterile flowers when grown on MS medium supplemented with sucrose							Seedling lethal with pale-green cotyledon and leaves. Plants are able to develop mature non-photosynthetic leaves thanks to the large reserves of maize seeds					
**CRP1 protein localization**								
*At*CRP1 is a component of plastid nucleoids and it is found associated to thylakoid membranes and in the stroma							*Zm*CRP1 has been reported to be highly enriched in plastid nucleoids and to localize exclusively in the chloroplast stroma					
**Thylakoid protein content**								
PSI		PSII			Cyt *b_6_f*	ATPase	PSI		PSII		Cyt *b_6_f*	ATPase
- (/PsaC)		-			/	-	-		=		/	=
**RNA targets**								
RIP-Chip		Slot-Blot			*In vivo* footprint		RIP-Chip		Slot-Blot		*In vivo* footprint	
*psaC*		*psaC*			*psaC*		*psaC*		*psaC*		n.r.	
*petB-petD*		*petB-petD*			*petB-petD*		/		/		*petB-petD*	
/		*petA*			*petA*		*petA*		*petA*		n.r.	
*rps15*		/			/		/		/		n.r.	
**Metabolism of chloroplast RNAs**								
	Accumulation			Processing defects			Accumulation			Processing defects		
*psaC*	-			No			=			No		
*petB-petD*	/			Yes			/			Yes		
*petA*	-			No			=			No		


*^a^Data are obtained from the present manuscript ^b^Data are obtained from Barkan et al. (1994, 2012), Fisk et al. (1999), Schmitz-Linneweber et al. (2005). -, marked reduction; /, complete absence; =, no changes; +, increase; n.r., not reported.*

### CRP1 Proteins Are Part of Chloroplast Nucleoids

We detected *At*CRP1 in the stroma and associated with thylakoid membranes (see **Figure 2**; **Table 1**), whereas *Zm*CRP1 is a stromal protein with no detectable association with chloroplast membranes (Fisk et al., 1999). The dual localization of *At*CRP1 within the chloroplast is supported by proteomic studies that detected *At*CRP1 in the grana-fraction of Arabidopsis thylakoids (Tomizioli et al., 2014) and in the stroma proteome, as part of Megadalton complexes (Olinares et al., 2010). In particular, *At*CRP1 appeared to be highly enriched in fractions that contained ribosomal proteins, translation factors, RNA helicases and other PPR proteins, suggesting a major role of *At*CRP1 in chloroplast gene expression. These data, together with the co-localization with GUN1 protein (see **Figure 2**), indicate that *At*CRP1 is integral to chloroplast nucleoids (Koussevitzky et al., 2007; Colombo et al., 2016; Tadini et al., 2016), i.e., the DNA-containing structures without defined boundaries that harbor the plastid gene expression machinery (Pfalz and Pfannschmidt, 2013; Melonek et al., 2016). Similarly, *Zm*CRP1 was found to be highly enriched in the nucleoid fractions of maize plastids, together with proteins involved in DNA replication, organization and repair as well as transcription, mRNA processing, splicing and editing (Majeran et al., 2012), further supporting the involvement of CRP1 proteins in plastid gene expression.

### CRP1 Proteins Are Required for the Biogenesis of the Photosynthetic Apparatus

The yellow-albinotic and seedling lethal phenotype exhibited by *atcrp1* is very similar to the chlorophyll deficient and lethal phenotype of *zmcrp1* plants (Barkan et al., 1994; Fisk et al., 1999). Arabidopsis mutants die at the two-cotyledon stage after germination on soil, but can overcome seedling lethality on sucrose-containing media, where they develop mature leaves and sterile flowers (see **Figure 4**; **Table 1**). Similarly, non-photosynthetic *zmcrp1* plants die at about 3 weeks after germination when seed reserves are exhausted. Furthermore, the *atcrp1* phenotype appears to be typical of Arabidopsis mutants lacking components of the photosynthetic apparatus and not of the gene expression machinery or of the protein import apparatus, since the latter usually result in the premature arrest at the globular-to-heart stage of embryo development, when chloroplast biogenesis begins (Ruppel and Hangarter, 2007; Romani et al., 2012; Beeler et al., 2014). Nevertheless, the pale-green pigmentation of the mutant embryo at bent-cotyledon stage (see **Figure 4**) and the β-glucuronidase (GUS) activity observed in young developing cotyledons and rosette leaves, but not in older tissues (see **Figure 5**), indicate that *AtCRP1* gene expression and protein accumulation is required during the very early stages of the photosynthetic apparatus assembly. Immunoblot data indicate, indeed, that *At*CRP1, like *Zm*CRP1, might act as a nuclear-encoded anterograde regulatory component responsible for coordination of the accumulation of Cyt *b_6_/f* and PSI protein complexes (see **Figure 6**). Besides their role in linear electron transport (LET), Cyt *b_6_/f* and PSI indeed play a key role in Cyclic Electron Transport (CET), which has been reported to be enhanced in Arabidopsis green seeds and to be required for optimal seed vigor and seed germination rate (Allorent et al., 2015).

In contrast to *zmcrp1* plants (Barkan et al., 1994), the absence of *At*CRP1 destabilized the entire photosynthetic apparatus, as shown by the marked reduction of PSII core, ATPase and LHC protein levels. The general down-regulation of thylakoid complexes owing to defects in the intersystem electron transport chain appears to be a common feature of Arabidopsis photosynthetic mutants and provides clear evidence of a different adaptive response between monocot and dicot plants (Meurer et al., 1996; Varotto et al., 2000, 2002; Maiwald et al., 2003; Weigel et al., 2003; Ihnatowicz et al., 2004, 2007; Belcher et al., 2015). Furthermore, the *atcrp1-1* phenotype, both in terms of plastid transcript and plastid protein accumulation, appears to be much more drastic than the one of other *ppr* mutants required for the processing and expression of *psbB-psbT-psbH-petB-petD* operon, such as *hcf152* (Meierhoff et al., 2003), suggesting that the absence of *At*CRP1 protein might affect the activity of other factors essential for plastid gene expression. As a matter of fact, rRNA abundance is markedly reduced in *atcrp1-1* plastids, indicating a general reduction of protein synthesis, as consequence of pleiotropic effects.

### RNA Targets: Commonalities and Divergences between *At*CRP1 and *Zm*CRP1 Proteins

RNA immunoprecipitation-Chip and slot blot data suggest a physical interaction between *At*CRP1 and the transcripts of *psaC. petB-petD* and possibly *petA*, even though it is not known whether these interactions are direct or mediated by other factors (see **Figure 7**). However, all of these RNAs harbor a region where a native footprint is annotated, raising the tempting hypothesis that *At*CRP1 is in fact the RNA-binding factor responsible for that footprint (see **Figure 8**). Furthermore, when these enriched fragments were searched for occurrences of the predicted binding motif of *At*CRP1, each of them proved to contain a hit inside the footprint region, strongly suggesting that *At*CRP1 could be the factor leaving those footprints. Nevertheless, the observation that the footprints identified in Arabidopsis *psaC, petA*, and *petB-petD* transcripts are larger than the 14 nucleotide size of the predicted *At*CRP1 footprint (37, 29, and 41 nucleotides in *psaC, petA*, and *petB-petD*, respectively) supports the view that the binding of *At*CRP1 to its targets *in vivo* could be stabilized by other protein partners. For instance, the peptide chain release factor B3 (PrfB3) has been also shown to be required for Arabidopsis autotrophic growth and for the stability of 3′ processed *petB* transcripts to adjust cytochrome *b_6_* levels (Stoppel et al., 2011), thus possibly being an *At*CRP1 specific protein partner. Similarly, PPR proteins involved in RNA stabilization and editing have been shown to interact with RNA Recognition Motif (RRM) proteins and other factors, indicating that larger protein complexes assembled around a PPR protein are likely to occur (Kupsch et al., 2012; Takenaka et al., 2014; Shi et al., 2015).

The interactions with the 5′UTR of *psaC* and *petA* have also been reported in the case of *Zm*CRP1 (Schmitz-Linneweber et al., 2005; Williams-Carrier et al., 2008), indicating that this feature of CRP1 function is conserved between Arabidopsis and maize. *Zm*CRP1 was also shown to bind directly to the 5′-UTR of *petA* transcripts by electrophoresis mobility shift assay (Williams-Carrier et al., 2008), favoring the possibility of a direct binding of CRP1 proteins to the corresponding RNA targets (see also **Figure 8**). Furthermore, *Zm*CRP1 has been proposed to directly control the translation of *petA* and *psaC* transcripts (Barkan et al., 1994), as shown through pulse labeling and polysome loading (in the case of *petA*), or deduced from the reduced association of *psaC* RNAs with ribosomes. Interestingly, the PsaC subunit of PSI and the PetA subunit of Cyt *b_6_/f* could not be detected in *atcrp1* thylakoids, despite the accumulation of the corresponding transcripts with no processing defects (see also **Figure 9**), suggesting that *At*CRP1 plays a major role in translation regulation also in Arabidopsis. Unfortunately, the specific requirement of *At*CRP1 in plastid protein translation cannot be verified by comparing Col-0 and *atcrp1-1* leaves, due to the marked reduction of *rRNA* accumulation in *atcrp1-1* plastids.

In addition to the defects in *petA* translation, the complete absence of Cyt *b_6_/f* protein complex observed in *atcrp1* thylakoids can also be attributed to processing alterations of the *psbB-psbT-psbH-petB-petD* polycistronic transcription unit. The lack of the monocistronic *petB*, the dicistronic *psbH-petB*, and the unspliced *petB* transcripts, together with the direct binding of *At*CRP1 to the *petB-petD* intergenic region, strongly support the role of *At*CRP1 in the metabolism of *petB* and *petD* transcripts. PPR protein-derived RNA-footprints are considered to arise due to exonucleolytic activity (Ruwe et al., 2016). Since sRNAs corresponding to predicted binding sites of *At*CRP1 are identified here, the most likely role for *At*CRP1 is to block exonucleases from degrading the *petB* and *petD* transcripts. A similar defect in *petB-petD* maturation has been reported in *zmcrp1* mutant plants (Barkan et al., 1994; Fisk et al., 1999; Schmitz-Linneweber et al., 2005), although no association was detected between *Zm*CRP1 and the *petB-petD* intergenic region (Schmitz-Linneweber et al., 2005), so it is still uncertain whether the role of *Zm*CRP1 is direct or indirect.

## Conclusion

Taken together, the characterization of the functional role of *At*CRP1 in chloroplast biogenesis has highlighted several features in common with the *Zm*CRP1. Both proteins appear to control, directly or indirectly, the expression of plastid genes encoding subunits of Cyt *b_6_/f* and PSI protein complexes. The coordination of the accumulation of these two protein complexes is fundamental to guarantee optimal photosynthesis in mature plants, but appears also to be important during seed germination, when cyclic electron transport is highly enhanced relative to LET.

Differences in RNA targets observed by immunoprecipitation and hybridization assays between *At*CRP1 and *Zm*CRP1 might be explained by a broad affinity for RNA targets, but may also have technical reasons (GFP antibody for Arabidopsis versus direct anti-ZmCRP1 antibody in maize). Evidence in favor of conservation of PPR protein activity between different species has been reported for the PLS and P subfamilies (Choury et al., 2005; Bolle and Kempken, 2006; Choury and Araya, 2006); for instance, the maize MPPR6 protein can complement loss-of-function Arabidopsis mutants lacking the orthologous protein (Manavski et al., 2012). However, functional divergence has been also observed, as in the case of orthologous PPR proteins ATP4 (maize) and SVR7 (Arabidopsis) (Liu et al., 2010; Zoschke et al., 2012, 2013a,b). Further studies aimed to verify the degree of protein activity conservation between monocots and dicots are needed to extend our knowledge of PPR protein functions and the degree of protein function conservation. The parallel characterization of PPR orthologs, including the relationship between their protein structures and the corresponding target RNA species, may represent an underestimated and powerful strategy to precisely determine the PPR code, essential for a fast and accurate large scale prediction of PPR targets, and for the functional characterization of the PPR-mediated nucleus-to-chloroplast anterograde signaling pathway.

## Author Contributions

RF, LT, FM, FR, SM, M-KL, CS-L, and PP participated to the organization of the manuscript. RF, LT, FM, FR, SM, MC, AC, and PP designed and carried out the experiments related to the molecular biology and biochemical characterization of atcrp1 mutants. RF, FM, LT, M-KL, and CS-L were involved in RIP-Chip and slot blot assays, as well as in the *in silico* identification of native footprints and prediction of AtCRP1 binding motif. PP wrote the manuscript.

## Conflict of Interest Statement

The authors declare that the research was conducted in the absence of any commercial or financial relationships that could be construed as a potential conflict of interest.

## Footnotes

1 http://www.plantppr.com

2 http://signal.salk.edu/cgi-bin/tdnaexpress

3 http://www.duchefa.com

4 https://fiji.sc/

5 http://www.agrisera.com/en/artiklar/plantalgal-cell-biology/index.html

6 http://www.thermofisher.com

7 http://meme-suite.org/tools/fimo

8 https://www.molgen.hu-berlin.de/projects-jbrowse-athaliana.php

9 http://www.maizegdb.org/

10 http://www.cbs.dtu.dk/services/ChloroP/

11 https://www.molgen.hu-berlin.de/projects-jbrowse-athaliana.php

12 http://meme-suite.org/tools/fimo

## Supplementary Material

**TABLE S1 | RNA immunoprecipitation (RIP-Chip) data.**

**TABLE S2 | Oligonucleotides employed for cloning, genotyping, northern blot, slot blot and qRT-PCR assays.**
